# Identification of Bradycardia Following Remdesivir Administration Through the US Food and Drug Administration American College of Medical Toxicology COVID-19 Toxic Pharmacovigilance Project

**DOI:** 10.1001/jamanetworkopen.2022.55815

**Published:** 2023-02-14

**Authors:** Jason M. Devgun, Rongmei Zhang, Jeffrey Brent, Paul Wax, Keith Burkhart, Alison Meyn, Sharan Campleman, Stephanie Abston, Kim Aldy

**Affiliations:** 1 Department of Emergency Medicine, Washington University School of Medicine, St Louis, Missouri; 2 Center for Drug Evaluation and Research Food and Drug Administration, Silver Spring, Maryland; 3 Department of Medicine, University of Colorado School of Medicine, Aurora; 4 American College of Medical Toxicology, Phoenix, Arizona; 5 Department of Emergency Medicine, The University of Texas Southwestern Medical Center, Dallas

## Abstract

**Importance:**

The rapid spread and mortality associated with COVID-19 emphasized a need for surveillance system development to identify adverse events (AEs) to emerging therapeutics. Bradycardia is a remdesivir infusion–associated AE listed in the US Food and Drug Administration–approved prescribing information.

**Objective:**

To evaluate the magnitude and duration of bradycardic events following remdesivir administration.

**Design, Setting, and Participants:**

A multicenter cohort study of patients with recorded heart rate less than 60 beats per minute within 24 hours after administration of a remdesivir dose was conducted between November 23, 2020, and October 31, 2021. Participants included patients hospitalized with COVID-19 at 15 medical centers across the US. Patients excluded had AEs unrelated to bradycardia, AEs in addition to bradycardia, or first onset of bradycardia after 5 remdesivir doses.

**Exposures:**

Remdesivir administration.

**Main Outcomes and Measures:**

Linear mixed-effect models for the minimum HR before starting remdesivir and within 24 hours of each dose included doses as fixed effects. Baseline covariates were age (≥65 years vs <65 years), sex (male vs female), cardiovascular disease history (yes vs no), and concomitant use of bradycardia-associated medications. The interactions between variables and doses were considered fixed-effects covariates to adjust models.

**Results:**

A total of 188 patients were included in the primary analysis and 181 in the secondary analysis. The cohort included 108 men (57.4%); 75 individuals (39.9%) were non-Hispanic White and mean (SD) age was 61.3 (15.4) years. Minimum HR after doses 1 to 5 was lower than before remdesivir. Mean minimum HR was lowest after dose 4, decreasing by −15.2 beats per minute (95% CI, −17.4 to −13.1; *P* < .001) compared with before remdesivir administration. Mean (SD) minimum HR was 55.6 (10.2) beats per minute across all 5 doses. Of 181 patients included in time-to-event analysis, 91 had their first episode of bradycardia within 23.4 hours (95% CI, 20.1-31.5 hours) and 91 had their lowest HR within 60.7 hours (95% CI, 54.0-68.3 hours). Median time to first bradycardia after starting remdesivir was shorter for patients aged 65 years or older vs those younger than 65 years (18.7 hours; 95% CI, 16.8-23.7 hours vs 31.5 hours; 95% CI, 22.7-39.3 hours; *P* = .04). Median time to lowest HR was shorter for men vs women (54.2 hours; 95% CI, 47.3-62.0 hours vs 71.0 hours; 95% CI, 59.5-79.6 hours; *P* = .02).

**Conclusions and Relevance:**

In this cohort study, bradycardia occurred during remdesivir infusion and persisted. Given the widespread use of remdesivir, practitioners should be aware of this safety signal.

## Key Points

**Question:**

For hospitalized patients with COVID-19 receiving remdesivir, is there an association between dose and time to bradycardia in those experiencing bradycardic events?

**Findings:**

In this cohort study, among 188 patients receiving remdesivir who had bradycardia, the most substantial decrease in heart rate occurred within 24 hours of the loading dose. The median time to minimum heart rate was 60.7 hours after remdesivir administration.

**Meaning:**

In this study, bradycardia in patients receiving remdesivir was seen during the infusion and after 1 or more doses; however, these findings are limited because a large proportion of patients had cardiac risk factors and/or severe COVID-19.

## Teaser

This cohort study examines the incidence of bradycardia occurring during or after administration of remdesivir in patients with COVID-19 through the US Food and Drug Administration American College of Medical Toxicology COVID-19 Toxic (FACT) Pharmacovigilance Project.

## Introduction

When the COVID-19 pandemic emerged, many treatments were used either off-label, under US Food and Drug Administration (FDA) emergency use authorizations, or through compassionate use programs. Several therapeutics proposed early in the pandemic had unproven benefit and potential for cardiovascular toxic effects.^1,2^ The rapid spread and high mortality of COVID-19 emphasized a need to develop a surveillance system to identify adverse events (AEs) specifically related to emerging COVID-19 therapeutics. In response, the American College of Medical Toxicology Toxicology Investigators Consortium (ToxIC)^3,4^ contracted with the FDA to establish a multicenter surveillance network to actively identify and characterize AEs associated with the treatment or prevention of COVID-19 in practice settings. The FDA ACMT COVID-19 Toxic (FACT) Pharmacovigilance Project was launched in September 2020 and is led by medical toxicology physician investigators from 15 major medical centers in 11 states and the District of Columbia.^5^

On May 1, 2020, the FDA issued an emergency use authorization for remdesivir use in patients hospitalized with severe COVID-19.^6^ Studies reported that remdesivir confers a reduction in hospitalization days, but not a mortality benefit, for patients with COVID-19.^7,8,9^ In prior trials of remdesivir use for treatment of Ebola and COVID-19, rare cases of hypotension and arrhythmias were noted^10,11^; however, bradycardia was not a recognized AE associated with remdesivir until its subsequent widespread use. Within the first month of implementation of the FACT project, we identified a potential safety signal of persistent or delayed remdesivir-associated bradycardia outside the infusion period and created a detailed data collection tool to investigate this occurrence. This study aimed to describe the magnitude and duration of bradycardic events following remdesivir administration and to identify potential risk factors for this AE.

## Methods

The FACT Pharmacovigilance Project is an active surveillance program with 15 participating US medical centers during the study period focused on identifying possible AEs, medication errors, toxic effects, and/or overdose related to any nonvaccine medication or substance administered with intent to treat or prevent COVID-19 infection. Between November 23, 2020, and October 31, 2021, deidentified cases were identified by research assistants and medical toxicology physician site investigators via site-specific mechanisms, including inpatient electronic health record (EHR) review, pharmacy inquiries, and discussions with treatment teams. This cohort study was reviewed by the Western Institutional Review Board (IRB) and each site’s local IRB prior to initiation. A waiver of informed consent was provided by the Western IRB for this medical records review project as non-human research. This investigation followed the Strengthening the Reporting of Observational Studies in Epidemiology (STROBE) reporting guideline.

Using a standardized data collection tool (REDCap), data from EHRs were gathered including demographic characteristics, case narrative, clinical signs and symptoms, and treatment and outcomes for AEs. Data regarding race and ethnicity were obtained to include as part of the demographic characteristics through the EHR with categories based on US Office of Management and Budget standards.^12^ ToxIC alerted sites regarding bradycardia associated with remdesivir outside the infusion period and created a data collection instrument that included vital signs, including during the infusion period, and medication administration records. Data were collected for patients who met case criteria defined as any recorded heart rate (HR) less than 60 beats per minute (bpm) occurring after the start of the remdesivir regimen. Minimum HRs before remdesivir and within 24 hours of each dose were collected. Resolution of bradycardia was defined as an HR greater than 60 bpm sustained for 8 hours or more. Cases were defined as serious based on the FDA regulatory definition.^13^ Patients were included in analysis if they met our case definition of an HR less than 60 bpm within 24 hours of a remdesivir dose, and the bradycardia data collection instrument was completed. Patients were excluded if they had AEs either unrelated to bradycardia or in addition to bradycardia, if they reported the first bradycardic event more than 24 hours after the 5 doses of remdesivir for which data were collected, or if the bradycardia data collection instrument was not completed.

### Statistical Analysis

Patient demographic characteristics were summarized by number and percentage. In our primary analysis, we used linear mixed-effect models to model the minimum HR before remdesivir and within 24 hours of each dose administered with doses as fixed effects. Baseline covariates were age (≥65 years vs <65 years), sex at birth (male vs female), and cardiovascular history (yes vs no). Interactions between these variables and doses were considered covariates to adjust and identify effect modifications. Cardiovascular history was defined as having risk factors for atherosclerotic cardiovascular disease, such as diabetes, hypertension, hyperlipidemia, coronary artery disease, or a past myocardial infarction.^14^ The administration of medications associated with bradycardia, including β-blockers, calcium channel blockers, or digoxin, 24 hours prior to each dose was also considered as a time-varying covariate. Different covariance matrix structures (compound symmetry and the first-order autoregressive AR[1]) were considered to account for within-patient correlation. The fixed effects in our final model were determined by the likelihood ratio test and the selection of the covariance matrix was determined by Akaike information criteria. In our secondary analysis, the Kaplan-Meier estimator was used to characterize the time to the first bradycardic event and time to the minimum HR. All statistical analyses were conducted in SAS, version 9.4 (SAS Institute Inc). PROC MIXED was used to conduct longitudinal analysis where the outcome of the minimum HR was considered as a linear variable. The means of minimum HRs before and after remdesivir administration at different doses and subgroups were estimated from the model with differences tested by *t* test. PROC LIFETEST was used to produce Kaplan-Meier estimators for the time to first bradycardia and time to minimum HR with differences tested by the Wilcoxon test. With 2-sided testing, the significance threshold for all tests was set at *P* = .05.

## Results

**Figure 1.**
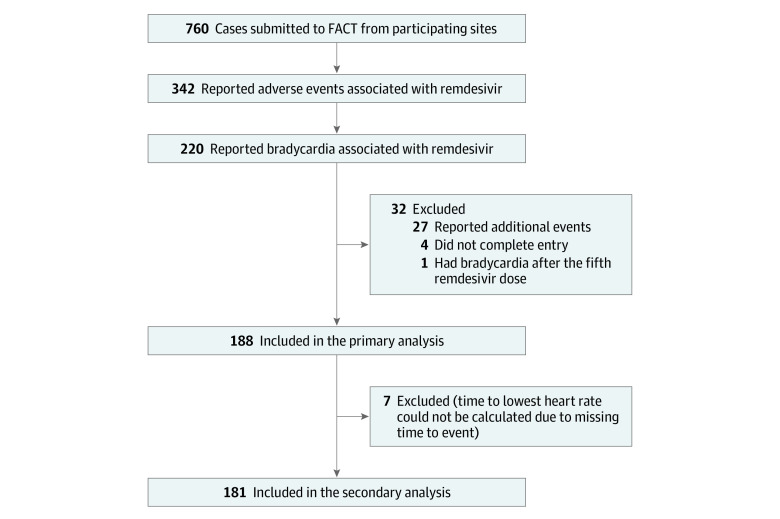
Flow Diagram for Included Cases FACT indicates the US Food and Drug Administration American College of Medical Toxicology COVID-19 Toxic Pharmacovigilance Project.

A total of 760 patients with AEs related to COVID-19 therapeutics were submitted to the FACT project between November 23, 2020, and October 31, 2021; 342 of these patients received remdesivir and 220 developed bradycardia after remdesivir administration. One hundred eighty-eight of these patients were included in the primary analysis and 181 in the secondary analysis, as outlined in Figure 1. Demographic characteristics of the cohort are included in Table 1. The cohort included 108 men (57.4%) and 80 women (42.6%), 75 individuals (39.9%) were non-Hispanic White, and mean (SD) age was 61 (15.4) years. Many patients had risk factors for severe illness with COVID-19,^15,16^ including 134 (71.3%) with a history of cardiac disease, 62 (33.0%) with diabetes, and 69 (36.7%) with obesity. Forty-two percent of the bradycardia cases were determined to be serious, with the most common reason being an HR 45 bpm or lower (36.7%). Other potentially serious AEs, such as QRS and/or QT prolongation (1.1%), sinus pauses (0.5%), ventricular tachycardia (0.5%), and cardiac arrest (1.1%), were rare; however, 5.3% of all patients required transition to a higher level of care due to the bradycardic event (Table 2). Five remdesivir doses were completed in 72.3% of the patients, with 4.8% receiving more than 5 doses due to critical illness or lack of improvement as recommended by the manufacturer, resulting in 22.9% of patients receiving less than 5 doses.^17^ Forty-five patients had infusion-associated bradycardia during at least 1 remdesivir dose (eTable 4 in Supplement 1). However, all 45 patients with a reported infusion-associated bradycardic episode had bradycardia continuing outside the infusion period.

**Table 1.** Demographic Characteristics and Comorbidities

Variable	No. (%) (N = 188)
Sex	
Men	108 (57.4)
Women	80 (42.6)
Age, mean (SD), y	61.3 (15.4)
Race	
Asian	4 (2.1)
Black/African American	51 (27.1)
Hispanic	56 (29.8)
Non-Hispanic White	75 (39.9)
Unknown	2 (1.1)
Comorbidities	
Healthy/no known comorbidities	18 (9.6)
Asthma/COPD	22 (11.7)
Diabetes	62 (33.0)
Immune-compromising condition (not HIV/AIDS)	5 (2.7)
HIV/AIDS	1 (0.5)
Obesity	69 (36.7)
Kidney disease^a^	21 (11.2)
Unknown past medical history	4 (2.1)
Cardiac history^b^	134 (71.3)
Arrhythmias	14 (7.4)
Atrial fibrillation	7 (3.7)
Atrial fibrillation and sick sinus syndrome	1 (0.5)
Unspecified	6 (3.2)
Medications associated with bradycardia prior to remdesivir^c^	
Amlodipine	21 (11.2)
Diltiazem	2 (1.1)
Verapamil	1 (0.5)
Metoprolol	17 (9.0)
Labetalol	3 (1.6)
Carvedilol	7 (3.7)
Amiodarone	3 (1.6)
Sotalol	1 (0.5)
Digoxin	1 (0.5)
Dexmedetomidine	9 (4.8)

Abbreviation: COPD, chronic obstructive pulmonary disease.

^a^ Kidney disease included chronic kidney disease and kidney failure.

^b^ Cardiac history included diabetes, hypertension, high cholesterol level, coronary artery disease, or a past myocardial infarction.

^c^ Listed as home medications before arrival or within 72 hours prior to first dose of remdesivir.

**Table 2.** Interventions for Bradycardia

Classification of adverse event	No. (%)^a^
Not serious	109 (58.0)
Serious	79 (42.0)
Potentially life-threatening^b^	73 (38.8)
HR ≤45 bpm	69 (36.7)
QRS (>120 ms) and/or QTc (>500 ms) prolongation	2 (1.1)
Sinus pause (≥3 s)	1 (0.5)
Ventricular tachycardia	1 (0.5)
Cardiac arrest	2 (1.1)
Interventions	
Stopped remdesivir	10 (5.3)
Temporary pacing	1 (0.5)
Atropine	3 (1.6)
Epinephrine	3 (1.6)
Norepinephrine	1 (0.5)
Dobutamine	2 (1.1)
Vasopressor NOS	1 (0.5)
Midodrine	1 (0.5)
Stopped β-blockers	1 (0.5)
Prolonged hospitalization	1 (0.5)
Transfer to stepdown unit	1 (0.5)
Transfer to ICU	9 (4.8)

Abbreviations: HR, heart rate; ICU, intensive care unit; NOS, not otherwise specified.

^a^ Percentages of the total cohort (n = 188).

^b^ One patient may have more than 1 potentially life-threatening serious event.

Remdesivir was discontinued due to bradycardia in 5.3% of the patients, and an intervention after the bradycardic event was reported in 34 patients (18.1%) (Table 2). Most bradycardic events occurred on inpatient floors (124 patients). There were 8 patients with bradycardic events recorded in the emergency department and 64 in the intensive care unit. For some patients, bradycardic events were recorded in multiple settings.

**Figure 2.**
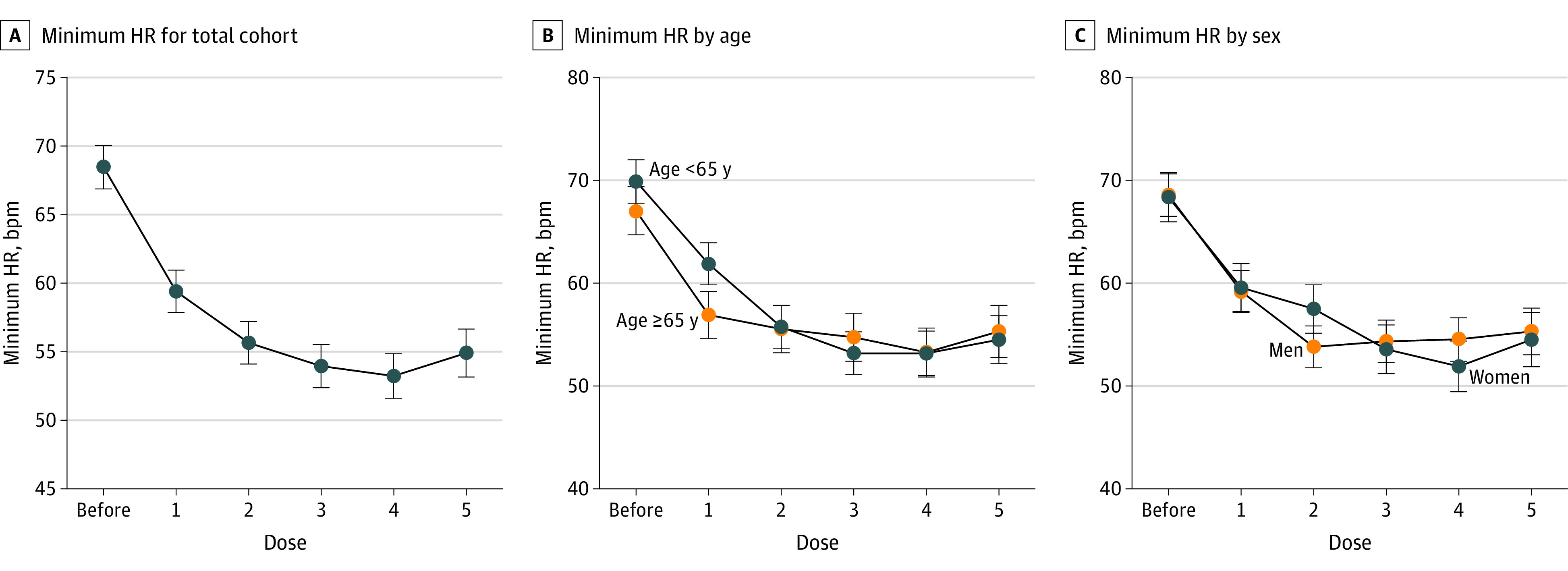
Minimum Heart Rate (HR) During Remdesivir Therapy Minimum HR shown for total cohort (A), in patients younger than 65 years vs 65 years or older (B), and in men vs women (C). Marginal mean minimum HR 48 hours prior to the first dose of remdesivir and 24 hours after each dose of remdesivir was determined using linear mixed-effect model. Error bars indicate 95% CI.

The final linear mixed-effect model included doses, age (≥65 years vs <65 years), sex at birth (male vs female), the interaction terms between doses and age, the interaction terms between doses and sex, and assumed AR(1) covariance matrix for repeated measures. Minimum HR was lower after doses 1 to 5 than before remdesivir (Figure 2A; eTable 1 in Supplement 1). Mean difference of the minimum HR between after dose 1 and before remdesivir was −9.1 bpm (95% CI, −10.6 to −7.6 bpm; *P* < .001). Mean minimum HR was reached after dose 4, where the mean difference between after dose 4 vs before remdesivir was −15.2 bpm (95% CI, −17.4 to −13.1 bpm; *P* < .001). Minimum HR after dose 1 was lower in patients aged 65 years or older than in those younger than 65 years, with a mean difference of −5.0 bpm (95% CI −8.0, −1.9 bpm; *P* = .002) (Figure 2B; eTable 2 in Supplement 1). Minimum HR after dose 2 for men was lower than for women, with mean difference −3.7 bpm (95% CI, −6.8 to −0.6 bpm; *P* = .02) (Figure 2C; eTable 3 in Supplement 1).

**Figure 3.**
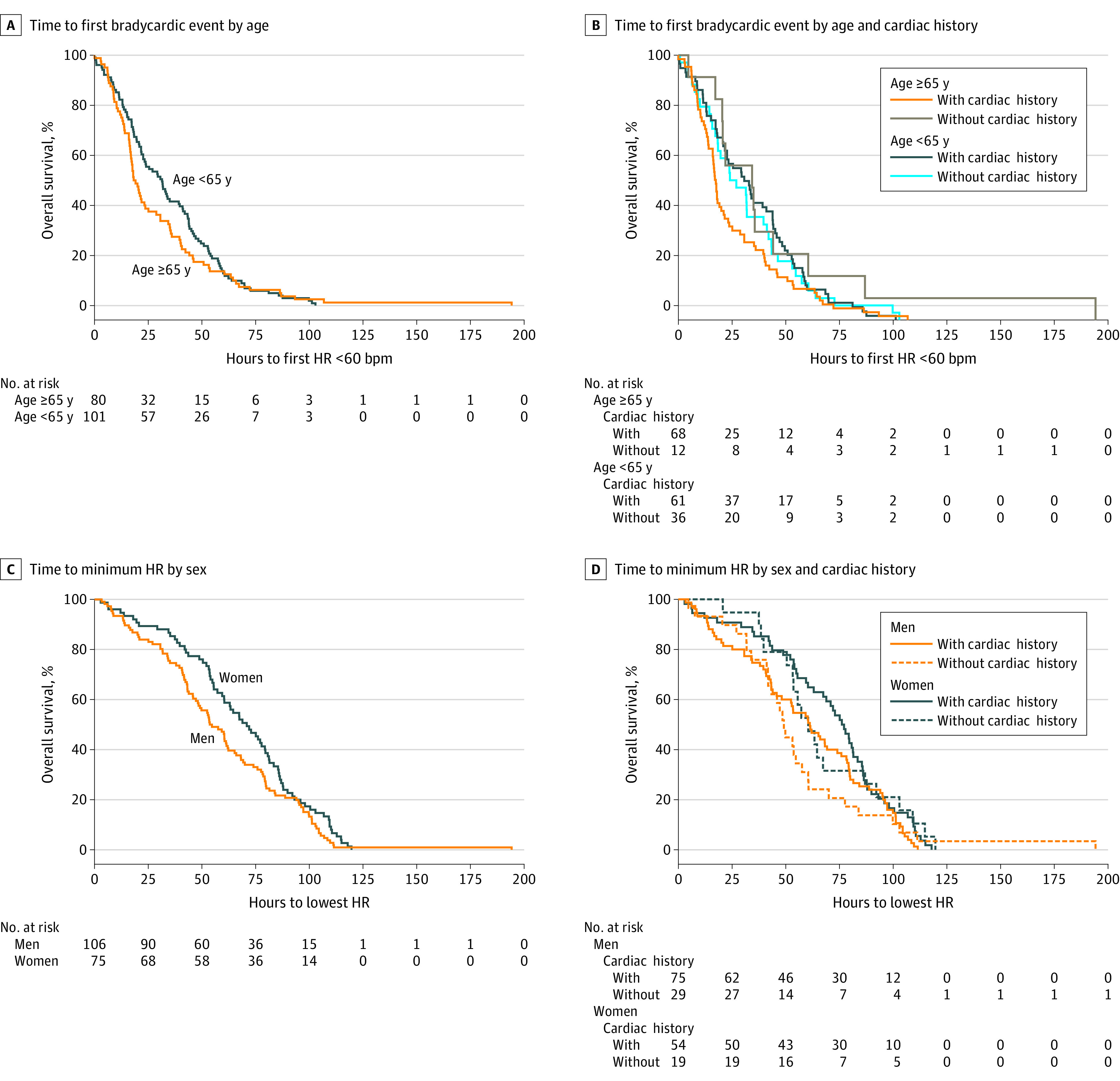
Time to Bradycardia by Age, Sex, and Cardiac History Time to first bradycardic event for patients younger than 65 years vs 65 years or older (Wilcoxon test of equality of survival curves by age group: χ^2^ = 4.0209; *P* = .04) (A) and patients younger than 65 years vs 65 years or older with and without a cardiac history (Wilcoxon test of equality of survival curves by group defined by age and cardiac history χ^2^ = 8.0097; *P* = .05) (B). Time to minimum HR for men vs women (Wilcoxon test of equality of survival curves by sex group: χ^2^ = 5.0721; *P* = .02; log-rank test of equality of survival curves by sex group: χ^2^ = 3.9275; *P* = .05) (C) and men vs women with and without a cardiac history (Wilcoxon test of equality of survival curves by group defined by sex and cardiac history: χ^2^ = 7.8979; *P* = .05) (D).

Time-to-event Kaplan-Meier plots were created for time to first bradycardic episode and time to the lowest HR. Of 181 patients included in time-to-event analysis, 91 experienced their first bradycardic episode within 23.4 hours (95% CI, 20.1-31.5 hours). Time to first bradycardic episode is shown in eFigure 1A in Supplement 1. Median time to first bradycardic episode was significantly shorter in patients aged 65 years or older compared with those younger than 65 years by the Wilcoxon test (18.7 hours; 95% CI, 16.8-23.7 hours vs 31.5 hours; 95% CI, 22.7-39.3 hours; *P* = .04) (Figure 3A). As shown in Figure 3B, the median time to the first bradycardic episode for patients aged 65 years or older with a cardiac history seemed shortest (17.7 hours; 95% CI, 16.1-21.6 hours) compared with other groups (*P* = .05, across 4 strata), but time to the first bradycardic episode was not significantly different with respect to the presence or absence of a cardiac history for the entire cohort (eFigure 1B in Supplement 1). There was no significant difference in the distribution of time to first bradycardic episode adjusted for sex (eFigure 1C in Supplement 1) or sex with the presence of a cardiac history (eFigure 1D in Supplement 1).

Mean (SD) minimum HR was 55.6 (10.2) bpm across all 5 doses. Ninety-one patients reached the lowest HR within 60.7 hours (95% CI, 54.0-68.3 hours) (eFigure 2A in Supplement 1). Median time to lowest minimum HR was significantly shorter for men than for women by the Wilcoxon test (54.2; 95% CI, 47.3-62.0 hours vs 71.0; 95% CI, 59.5-79.6 hours; *P* = .02) (Figure 3C). Time to lowest HR was still significantly different by sex accounting for the presence or absence of cardiac history by the Wilcoxon test (*P* = .05, across 4 strata) (Figure 3D). Among patients with a cardiac history, the median time to lowest HR for men was 61.3 hours (95% CI, 45.9-74.0 hours) and for women was 76.7 hours (95% CI, 63.1-81.5 hours). Among patients without a cardiac history, median time to lowest HR for men was 49.2 hours (95% CI, 41.7-57.6 hours) and for women was 60.5 hours (95% CI, 50.5-86.9 hours). Time to lowest HR was not significantly different when considering only age (χ^2^ = 1.22; *P* = .27), only cardiac history (χ^2^ = 1.61; *P* = .20), or age and cardiac history (χ^2^ = 4.72; *P* = .19) (eFigure 2B-D in Supplement 1).

## Discussion

In this cohort of patients with bradycardic events following remdesivir administration, we found these events to persist past the initial infusion, with the heart rate nadir typically occurring after the first dose with a median time to nadir of 60.7 hours. The median onset to a bradycardic event was within 24 hours for the total cohort and shorter for those aged 65 years and older and men. Serious AEs requiring intervention, such as arrhythmias, were rare; however, HRs less than or equal to 45 bpm were relatively common.

Remdesivir is a monophosphate prodrug converted to an active cyano-adenosine nucleoside analogue (GS-441524) and a metabolite resembling adenosine triphosphate (GS-443902) originally developed as a candidate drug for treatment of hepatitis C virus.^18^ The primary mechanism of action of remdesivir occurs in GS-443902 inhibition of RNA-dependent RNA polymerase resulting in premature termination of RNA synthesis.^19^ Although plasma concentrations of GS-441524 have an elimination half-life on the order of 1 to 2 hours, intracellular elimination may have an elimination half-life of 24 hours or more.^20^

Although bradycardia had not been reported as an AE associated with remdesivir prior to use in treatment of COVID-19, other RNA-dependent RNA polymerase inhibitors have been associated with cardiotoxicity, including bradycardia.^21,22^ Serious cardiovascular AEs are rarely reported for remdesivir in clinical trials for Ebola virus and COVID-19. A single episode of hypotension was reported in the initial clinical trial for Ebola virus of 175 patients; however, no information was provided regarding the timing of the event.^10^ The ACTT-1 trial for COVID-19 had 4 cases of unspecified arrhythmias in the remdesivir group (n = 532) vs 1 case of arrhythmia in the placebo group (n = 516).^23^ Other cardiac events reported in this trial included atrial fibrillation (5 cases in the remdesivir group vs 10 cases in the placebo group) and supraventricular tachycardia (3 cases vs 2 cases); however, neither of these differences was statistically significant. Bradycardia was not reported as an AE in this trial. Infusion-related reactions, including bradycardia, are listed in the package insert.^17^ In our cohort, infusion-related bradycardia was indicated during at least 1 remdesivir dose in 23.9% of the patients; however, 100% of patients with a reported infusion-associated bradycardic episode had continued bradycardia outside of the infusion period.

While cardiovascular events have been described from World Health Organization and FDA adverse event reporting system databases,^24,25,26^ and several individual case reports have described bradycardia associated with remdesivir within 2 to 5 days of treatment,^27,28,29,30,31^ the FACT Pharmacovigilance Project is one of the only large multicenter cohorts with HR data at each administration of remdesivir. Of the case reports that provided HR, 2 reported daily HR^29,30^ and 1 case report of a single patient provided multiple HR values.^27^ A small, single-center prospective study found a significant decrease in diurnal HR of patients treated with remdesivir compared with controls at days 4 and 5 of treatment.^32^ A small, single-center retrospective study found a significantly higher incidence of bradycardia after treatment day 3 of those treated with remdesivir.^33^ Although minimum HR was not specifically provided in these prior reports, our data of mean minimum HR (Figure 2A) are congruent with these reports and may indicate accumulation of GS-441524 or other cardioactive metabolites.

Due to variability in the frequency of documented vital signs across sites and levels of care, we chose time to first bradycardic episode and time to minimum HR as targets for time-to-event analysis. Although detailed vital sign trends are often not available in case reports of bradycardia following remdesivir, bradycardia was noted in several case reports after the second to fourth remdesivir dose in 19 cases including 5 cases in children.^27,28,29,30,31,34,35,36,37,38,39^ Three cases reported bradycardia within 24 hours of the 200-mg loading dose.^40,41,42^ In a single-center retrospective cohort of 473 patients, the incidence of bradycardia was higher after the third to fifth doses than after the first 2 doses.^43^ We found a median time to the first bradycardic episode of 23.4 hours. It is possible that in previous reports in the literature bradycardia was not noted until it was more pronounced. Still, a substantial number of patients had their time to first bradycardic episode in doses 2 to 5. The time to the first bradycardic episode was significantly shorter for patients aged 65 years or older. The reasons for this are unclear and may be multifactorial. While remdesivir is relatively quickly converted to active metabolites, GS-441524 is primarily eliminated in the urine.^17^ Decreased kidney clearance could result in accumulation of this metabolite and potentially explain this observed difference.

Several individual case reports of bradycardia following remdesivir administration indicate a minimum HR between 2 and 4 days, which is consistent with our modeled data (Figure 2A).^27,29,30,31,44^ A report of 2 cases of bradycardia following remdesivir had a minimum HR at day 5; however, the HR was only reported every 48 hours.^39^ A delayed onset to minimum HR fits with the relatively long elimination half-life of GS-441524 (22-29 hours) and other metabolites that may result in accumulation and ongoing effects.^45^ Men reached a minimum HR sooner than women. The reason for this is unclear; however, the difference may be due to variations in baseline resting HR among men and women as well as differences in baroreceptor response.^46,47^

We found that few patients (18.1%) in our cohort had an intervention. Three patients required atropine; unfortunately, sufficient detail was not available within the data to determine the response. Shirvani et al^31^ describe a series of 3 patients with bradycardia: 2 were unresponsive to atropine and 1 required a transvenous pacemaker. Jacinto et al^40^ describe a case of severe bradycardia unresponsive to atropine; the patient required dopamine, which was continued through the 5-day course and was promptly discontinued after the last dose. Temporary pacing was required in 1 patient with a history of heart failure with reduced ejection fraction and 3.7% of the patients required vasopressors that were believed to be related to remdesivir. In a prospective cohort of 100 patients with COVID-19 treated with remdesivir, there were no events requiring pacing and no episodes of hemodynamic instability or ventricular arrhythmias.^32^ Discontinuation of remdesivir before the recommended 5-day course due to bradycardia was the most common intervention in 5.3% of our patients and has been noted in other cases in the literature.^28,29,31,34,36,37,38,39^ As in the experience with these case reports, most cases of bradycardia were temporary and resolved with discontinuation of the medication. Even within the context of our pharmacovigilance project specifically targeting AEs, the overall incidence of serious AEs, such as those associated with atropine, pacing, or vasopressors, was low.

## Limitations

Our study has several limitations. First, the retrospective nature and focus on cases with identified AEs introduce selection bias. However, cases were actively identified and most cases occurred while the study was ongoing. The total number of patients with COVID-19 or subset of those treated with remdesivir across the 15 sites was not obtained. As such, we did not have a control group in this analysis of those receiving remdesivir without any bradycardic episodes. Our population included a high proportion (71.3%) of patients with comorbidities that may predispose them to bradycardic events. Although trained research assistants and investigators reported cases, the identification and review of cases varied based on the EHR and search strategy used at those sites. This report focuses on bradycardic events associated with remdesivir; however, several confounders, such as severe COVID-19 illness, hypoxia, and medication effects, may contribute to these bradycardic events. Although remdesivir was discontinued in only 5.3% of the patients due to bradycardia, 22.9% did not complete 5 or more doses. Due to this, each dose included fewer patients for analysis.

## Conclusions

In our cohort study, bradycardia following remdesivir infusion was observed directly after the infusion and throughout the course of treatment. Serious AEs requiring an intervention were rare. Further observational studies are needed to document the incidence of remdesivir-associated bradycardia and the relative risk for potentially vulnerable subgroups. Given the widespread use of remdesivir in COVID-19, practitioners should be aware of the potential for delayed-onset or sustained bradycardia. Most patients with bradycardia continued remdesivir therapy and had a good outcome.

## Notes

Supplement 1. **eTable 1.** Marginal Mean Minimum HR Before and After Each Remdesivir Dose, and Comparisons of Minimum HR Between Doses **eTable 2.** Marginal Mean Minimum HR Before and After Each Remdesivir Dose by Age Group **eTable 3.** Marginal Mean Minimum HR Before and After Each Remdesivir Dose by Sex **eTable 4.** Infusion-Associated Bradycardia Compared With the Remdesivir Dose Associated With the Minimum HR **eFigure 1.** Time to First Bradycardic Event for Total Cohort, Cardiac History, Sex, and Sex and Cardiac History **eFigure 2.** Time to Lowest HR Observed for Total Cohort, Age, Cardiac History, and Age and Cardiac History

Supplement 2. **Nonauthor Collaborators.** Toxicology Investigators Consortium FACT Study Group

Supplement 3.
Data Sharing Statement
