# Supplementary material for: Identification of Bradycardia Following Remdesivir Administration Through the US Food and Drug Administration American College of Medical Toxicology COVID-19 Toxic Pharmacovigilance Project
Source: JAMA Netw Open. 2023 Feb 14;6(2):e2255815. doi: 10.1001/jamanetworkopen.2022.55815 (PMC9929701; doi:10.1001/jamanetworkopen.2022.55815)
Supplement: Supplement 1. — eTable 1. Marginal Mean Minimum HR Before and After Each Remdesivir Dose, and Comparisons of Minimum HR Between Doses eTable 2. Marginal Mean Minimum HR Before and After Each Remdesivir Dose by Age Group eTable 3. Marginal Mean Minimum HR Before and After Each Remdesivir Dose by Sex eTable 4. Infusion-Associated Bradycardia Compared With the Remdesivir Dose Associated With the Minimum HR eFigure 1. Time to First Bradycardic Event for Total Cohort, Cardiac History, Sex, and Sex and Cardiac History eFigure 2. Time to Lowest HR Observed for Total Cohort, Age, Cardiac History, and Age and Cardiac History [file jamanetwopen-e2255815-s001.pdf]

Supplementary Online Content

Devgun JM, Zhang R, Brent J, et al; Toxicology Investigators Consortium FACT Study Group. Identification of bradycardia following remdesivir administration through the US Food and Drug Administration American College of Medical Toxicology COVID-19 Toxic Pharmacovigilance Project. *JAMA Netw Open*. 2023;6(2):e2255815. doi:10.1001/jamanetworkopen.2022.55815

eTable 1. Marginal Mean Minimum HR Before and After Each Remdesivir Dose, and Comparisons of Minimum HR Between Doses

eTable 2. Marginal Mean Minimum HR Before and After Each Remdesivir Dose by Age Group

eTable 3. Marginal Mean Minimum HR Before and After Each Remdesivir Dose by Sex

eTable 4. Infusion-Associated Bradycardia Compared With the Remdesivir Dose Associated With the Minimum HR

eFigure 1. Time to First Bradycardic Event for Total Cohort, Cardiac History, Sex, and Sex and Cardiac History

eFigure 2. Time to Lowest HR Observed for Total Cohort, Age, Cardiac History, and Age and Cardiac History

This supplementary material has been provided by the authors to give readers additional information about their work.

© 2023 Devgun JM et al. *JAMA Network Open.*

eTable 1: Marginal mean minimum HR before and after each remdesivir dose, and comparisons of minimum HR between doses.

	Mean (SD)	Difference vs. Before (95% CI)	Min HR Difference vs. Dose 1 (95% CI)	Difference vs. Dose 2 (95% CI)	Difference vs. Dose 3 (95% CI)	Difference vs. Dose 4 (95% CI)
**Before**	68.5 (10.7)					
**Dose 1**	59.5 (10.8)	-9.1 (-10.6, -7.6)* *P<0.0001*				
**Dose 2**	55.7 (10.8)	-12.8 (-14.7, -11.0)* *P<0.0001*	-3.8 (-5.2, -2.3)* *P<0.0001*			
**Dose 3**	54.0 (10.7)	-14.5 (-16.6, -12.5)* *P<0.0001*	-5.4 (-7.3, -3.6)* *P<0.0001*	-1.7 (-3.2, -0.2)* *P=0.0255*		
**Dose 4**	53.2 (10.6)	-15.2 (-17.4 -13.1)* *P<0.0001*	-6.2 (-8.2, -4.1)* *P<0.0001*	-2.4 (-4.3, -0.5)* *P=0.0115*	-0.7 (-2.3, 0.8) *P=0.3461*	
**Dose 5**	54.9 (10.5)	-13.6 (-15.9, -11.3)* *P<0.0001*	-4.5 (-6.7, -2.2)* *P<0.0001*	-0.7 (-2.9, 1.4) *P=0.4928*	0.9 (-1.0, 2.9) *P=0.3525*	1.7 (-0.0, 3.3) *P=0.0454*

*: $p<0.05$; mean, SD, and minimum HR between difference doses and corresponding 95% CI were estimated from the linear mixed model

© 2023 Devgun JM et al. *JAMA Network Open.*

eTable 2: Marginal mean minimum HR before and after each remdesivir dose by age group.

	Min HR		
	**≥65 years old Mean (SD)**	**<65 years old Mean (SD)**	**Difference ≥65 vs. <65 (95% CI)**
**Before**	67.1 (10.5)	69.9 (10.6)	-2.8 (-6.0, 0.3)<br>*P*=0.0758
**Dose 1**	56.9 (10.6)	61.9 (10.7)	-5.0 (-8.0, -1.9)*<br>*P*=0.0016
**Dose 2**	55.6 (10.6)	55.8 (10.7)	-0.2 (-3.3, 2.9)<br>*P*=0.8988
**Dose 3**	54.7 (10.5)	53.2 (10.6)	1.6 (-1.6, 4.7)<br>*P=0.3264*
**Dose 4**	53.3 (10.5)	53.2 (10.5)	0.1 (-3.1, 3.3)<br>*P=0.9538*
**Dose 5**	55.3 (10.4)	54.5 (10.4)	0.8 (-2.6, 4.2)<br>*P=0.6443*

*: $p<0.05$; mean, SD, and minimum HR between difference doses and corresponding 95% CI were estimated from the mixed model

© 2023 Devgun JM et al. *JAMA Network Open.*

eTable 3: Marginal mean minimum HR before and after each remdesivir dose by sex.

	Min HR<br>Male<br>Mean (SD)	Female<br>Mean (SD)	Difference<br>Male vs. Female (95% CI)
**Before**	68.6 (10.6)	68.4 (10.5)	0.2 (-3.0, 3.4)<br>*P=0.8986*
**Dose 1**	59.2 (10.6)	59.6 (10.6)	-0.4 (-3.4, 2.7)<br>*P=0.8190*
**Dose 2**	53.8 (10.6)	57.5 (10.6)	-3.7 (-6.8, -0.6)*<br>*P=0.0198*
**Dose 3**	54.4 (10.5)	53.6 (10.6)	0.8 (-2.3, 3.9)<br>*P=0.6243*
**Dose 4**	54.5 (10.5)	51.9 (10.5)	2.6 (-0.6, 5.9)<br>*P=0.1114*
**Dose 5**	55.3 (10.3)	54.5 (10.3)	0.8 (-2.7, 4.3)<br>*P=0.6471*

*: $p<0.05$; mean, SD, and minimum HR between difference doses and corresponding 95% CI were estimated from the mixed model

© 2023 Devgun JM et al. *JAMA Network Open.*

eTable 4: Infusion-associated bradycardia compared with the remdesivir dose associated with the minimum HR.

	Remdesivir dose with overall minimum HR*					
**Bradycardia during infusion**	1	2	3	4	5	**Total**
During 1st remdesivir infusion	**6**	0	1	4	0	11
During 2nd remdesivir infusion	2	**2**	5	2	1	12
During 3rd remdesivir infusion	2	0	**6**	5	1	14
During 4th remdesivir infusion	2	0	4	**12**	3	21
During 5th remdesivir infusion	1	0	2	7	**4**	14
**Total reported bradycardia during infusion**						**72**

*Note cases may have more than one remdesivir dose associated with bradycardia during infusion. n=45 cases reported bradycardia during an infusion

© 2023 Devgun JM et al. *JAMA Network Open.*

eFigure 1a-d: Time to first bradycardic event for a) total cohort, b) cardiac history (±card hx), c) sex (M, F), d) sex and cardiac history

a)

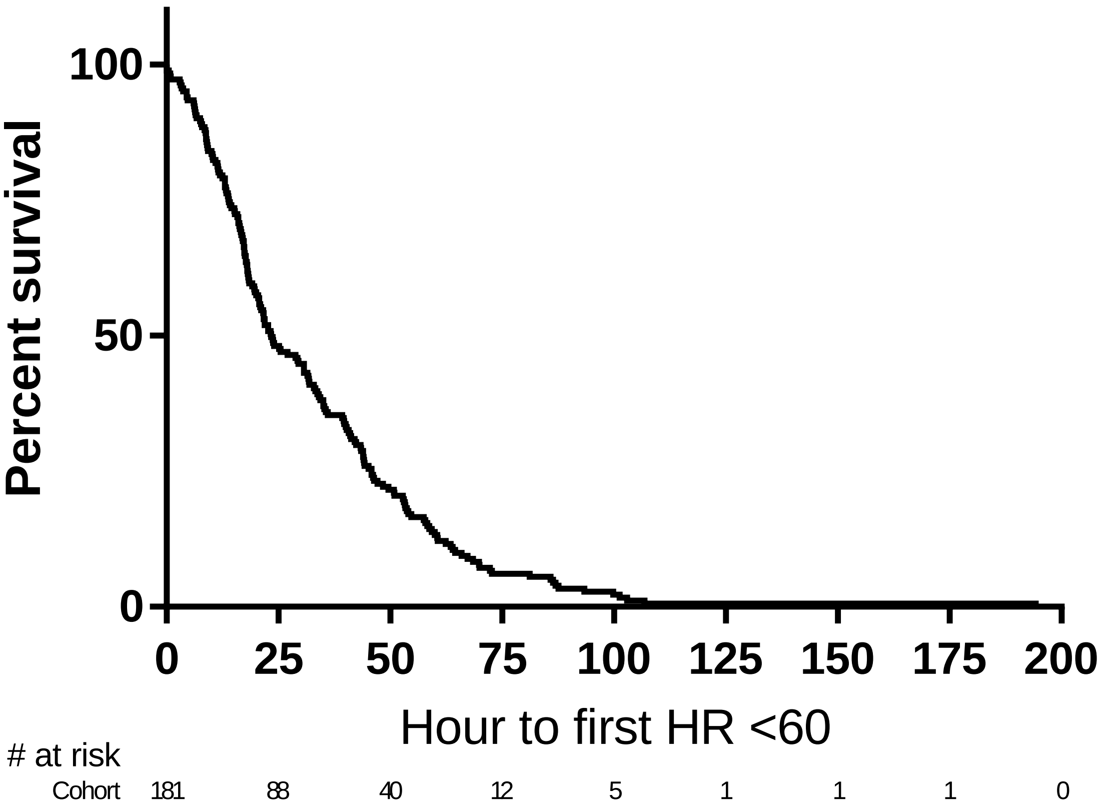

© 2023 Devgun JM et al. *JAMA Network Open.*

b)

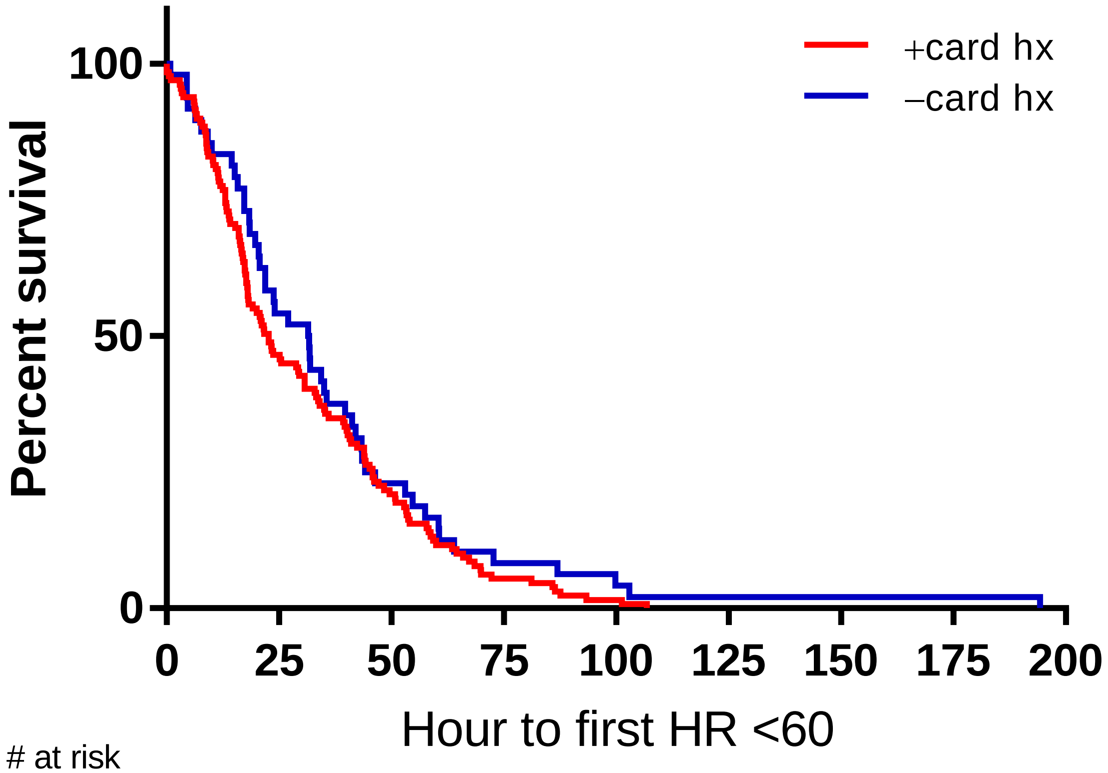

at risk

+card hx	129	61	28	8	3	0	0	0	0
–card hx	48	27	12	5	3	1	1	1	0

Wilcoxon test of equality of survival curves by with vs. without cardiac history, χ^2=1.1208, *p*=0.2897

© 2023 Devgun JM et al. *JAMA Network Open.*

c)

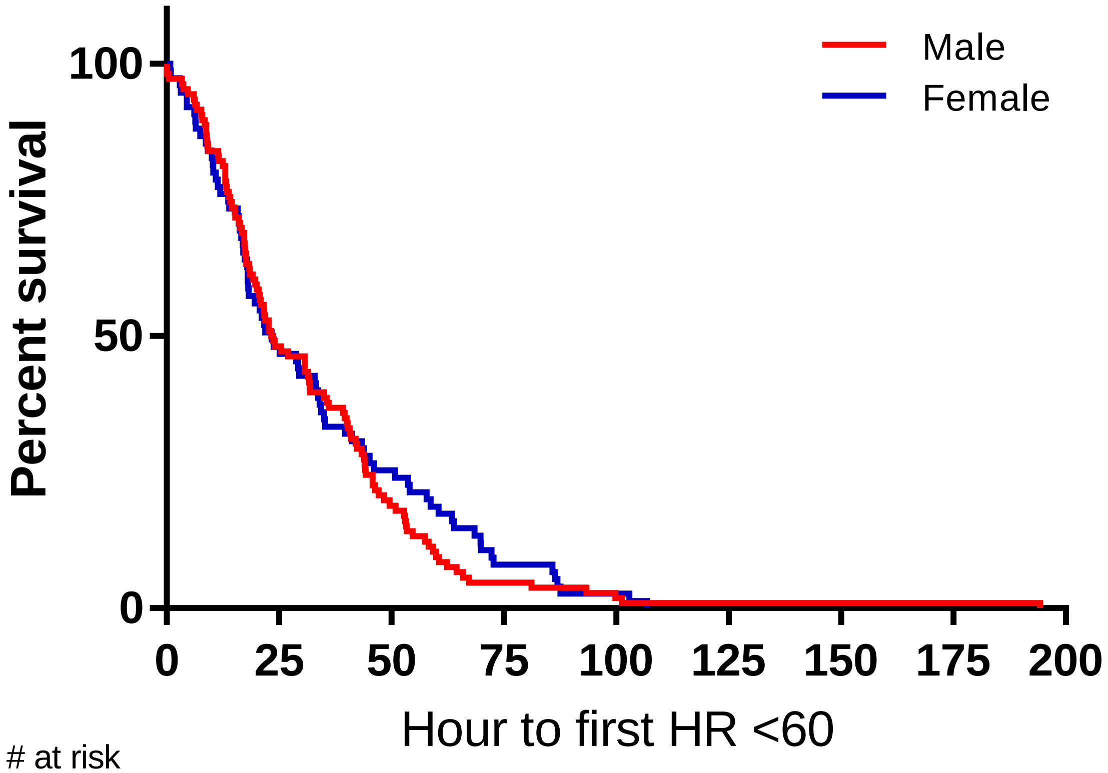

# at risk									
Male	106	52	21	6	3	1	1	1	0
Female	75	37	20	7	3	0	0	0	0

Wilcoxon test of equality of survival curves by male vs. female, χ^2=0.0044, *p*=0.9472

© 2023 Devgun JM et al. *JAMA Network Open.*

d)

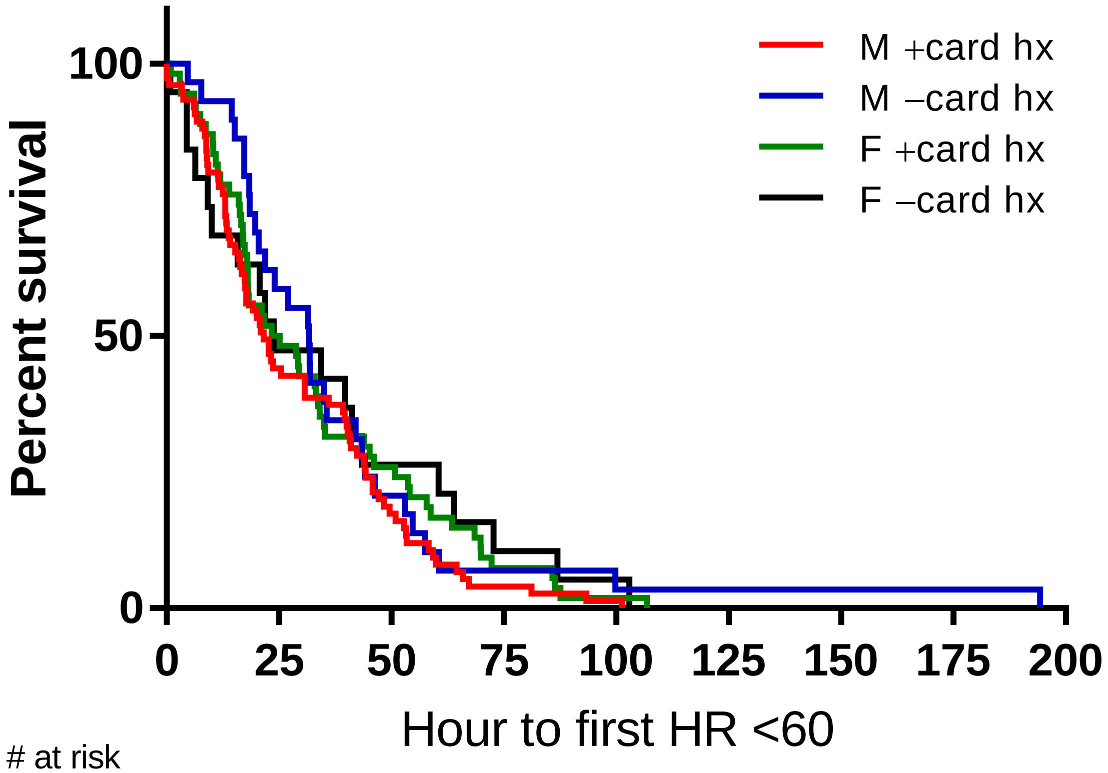

at risk

M +card hx	75	34	14	4	2	0	0	0	0
M –card hx	29	18	7	3	2	1	1	1	0
F +card hx	54	28	15	5	2	0	0	0	0
F +card hx	19	10	6	3	2	0	0	0	0

Wilcoxon test of equality of survival curves by four groups defined by sex and cardiac history, χ^2=2.0956, *p*=0.5528

© 2023 Devgun JM et al. *JAMA Network Open.*

eFigure 2a-d: Time to lowest HR observed for a) total cohort, b) age, c) cardiac history (±card hx), and d) age and cardiac history

a)

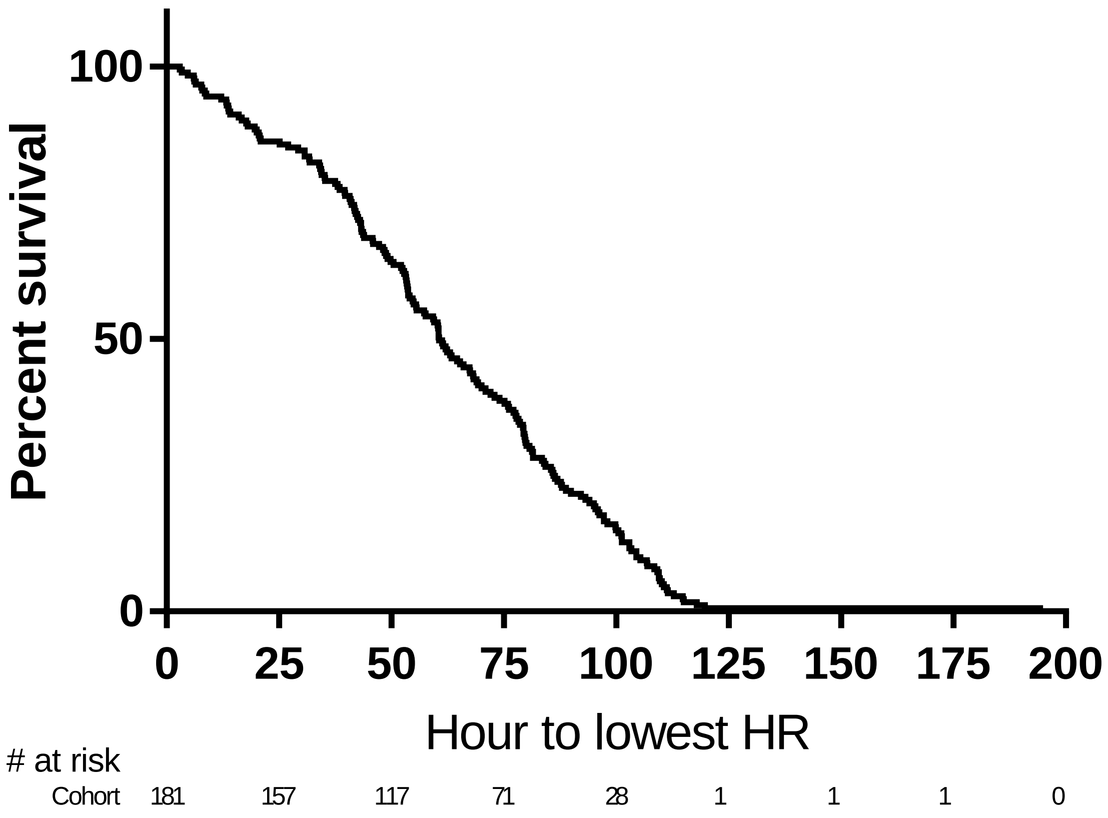

© 2023 Devgun JM et al. *JAMA Network Open.*

b)

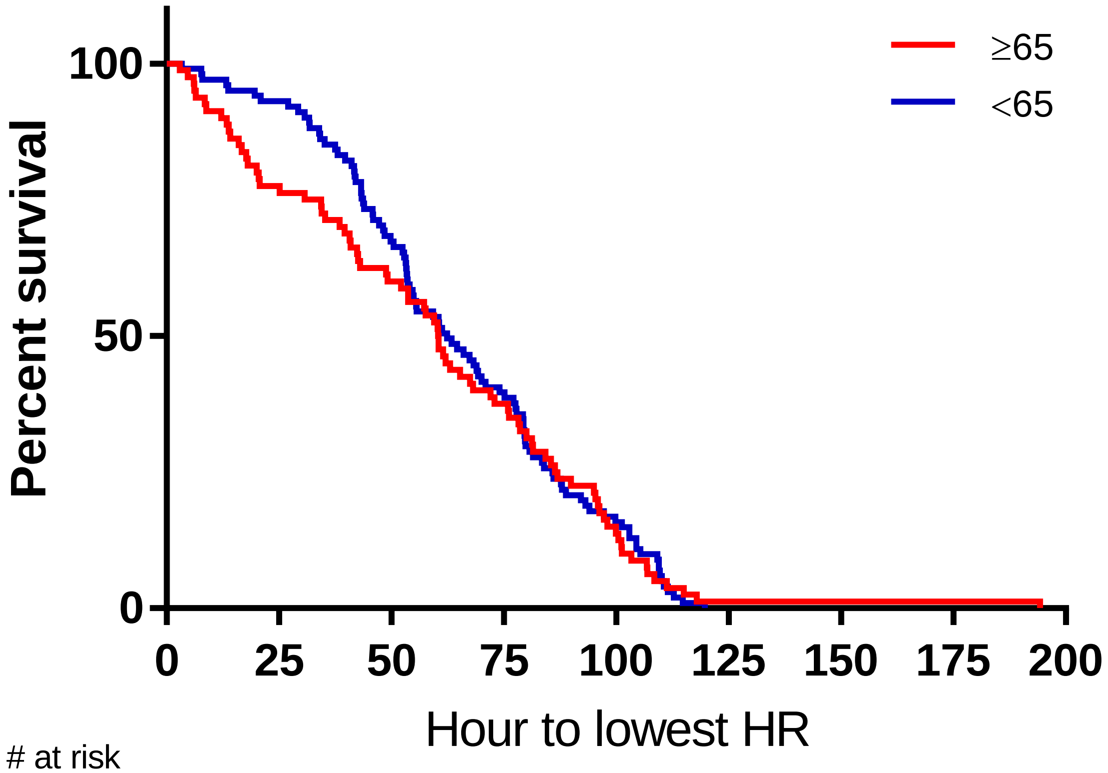

at risk

≥65	80	63	49	31	12	1	1	1	0
<65	101	95	69	41	17	0	0	0	0

Wilcoxon test of equality of survival curves by age, χ^2=1.2227, *p*=0.2688.

© 2023 Devgun JM et al. *JAMA Network Open.*

c)

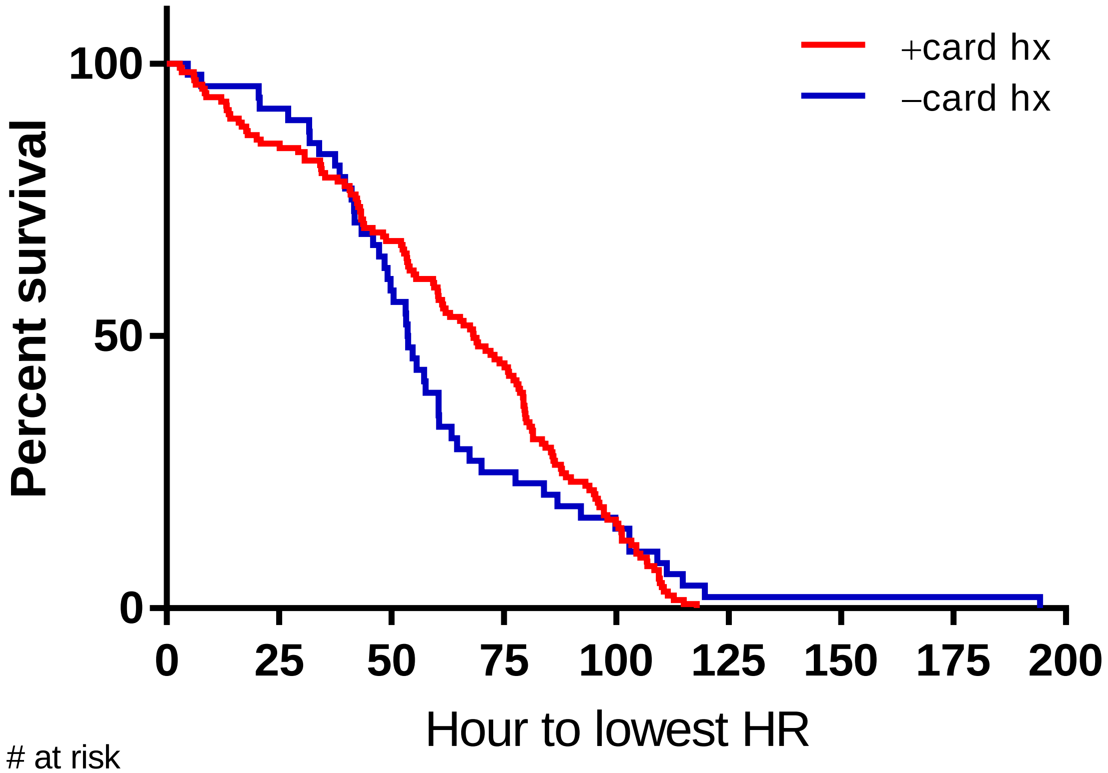

# at risk									
+card hx	129	111	88	59	21	0	0	0	0
–card hx	48	45	29	13	8	1	1	1	0

Wilcoxon test of equality of survival curves by with vs. without cardiac history, χ^2=1.6108, *p*=0.2044

© 2023 Devgun JM et al. *JAMA Network Open.*

d)

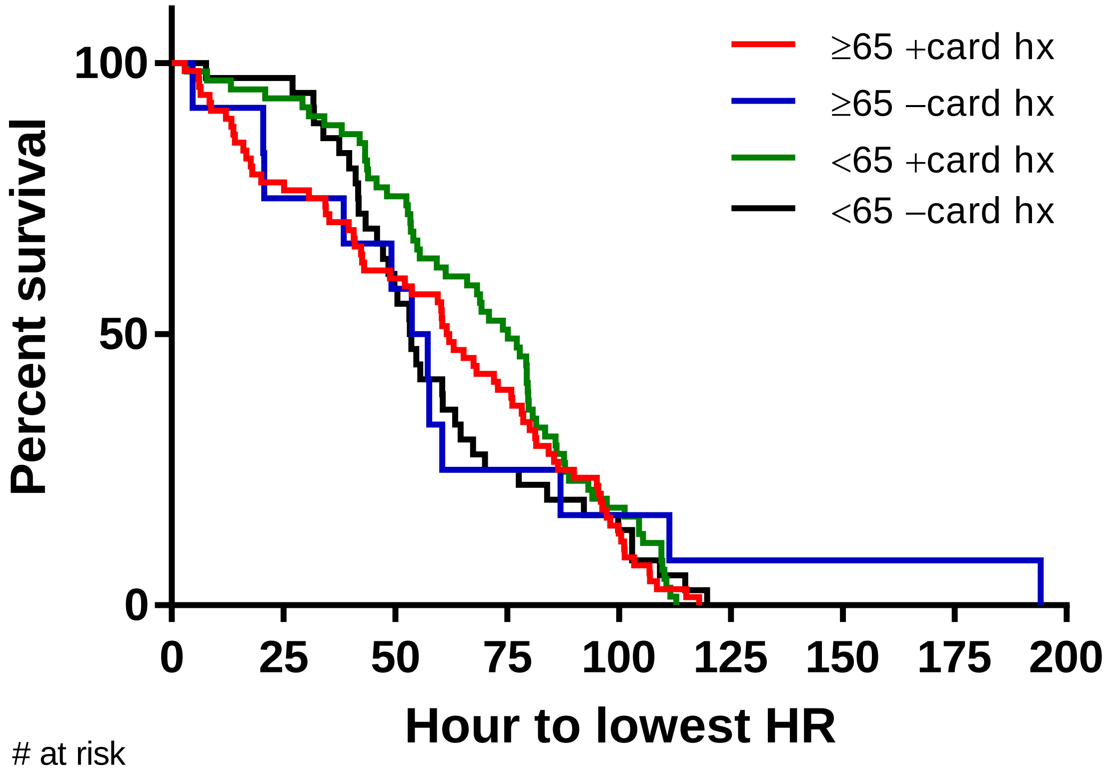

# at risk									
≥65 +card hx	68	54	42	28	10	0	0	0	0
≥65 –card hx	12	10	9	4	3	1	1	1	0
<65 +card hx	61	58	47	32	12	0	0	0	0
<65 –card hx	36	36	23	10	6	0	0	0	0

Wilcoxon test of equality of survival curves by four groups defined by age and cardiac history, χ^2=4.7227, *p*=0.1933

© 2023 Devgun JM et al. *JAMA Network Open.*
