# Supplementary material for: Identification of Bradycardia Following Remdesivir Administration Through the US Food and Drug Administration American College of Medical Toxicology COVID-19 Toxic Pharmacovigilance Project
Source: JAMA Netw Open. 2023 Feb 14;6(2):e2255815. doi: 10.1001/jamanetworkopen.2022.55815 (PMC9929701; doi:10.1001/jamanetworkopen.2022.55815)
Supplement: Supplement 2. — Nonauthor Collaborators. Toxicology Investigators Consortium FACT Study Group [file jamanetwopen-e2255815-s002.pdf]

Supplemental Online Content: Nonauthor Collaborators
*First name, last name, and suffix (if applicable) are required and will appear in PubMed.

*Group Name(s): Toxicology Investigators Consortium FACT Study Group							
***First Name and Middle Initial(s)**	***Last Name**	***Suffix (eg, Jr, III)**	Academic Degrees	Institution	Location (city, state/province, country)	Role or Contribution, eg, chair, principal investigator	Group (if more than 1 Group listed in the byline) and/or Subgroup (eg, Steering Committee)
Maryann	Amirshahi		MD	MedStar Washington Hospital	Washington, DC, USA	Site Investigator	Toxicology Investigators Consortium FACT Study Group
Katherine	Boyle		MD	Beth Israel Hospital	Boston, MA, USA	Site Investigator	Toxicology Investigators Consortium FACT Study Group
Jennie	Buchanan		MD	Denver Health Medical Center	Denver, CO, USA	Site Investigator	Toxicology Investigators Consortium FACT Study Group
Jennifer	Carey		MD	University of Massachusetts Memorial Medical Center	Worcester, MA, USA	Site Investigator	Toxicology Investigators Consortium FACT Study Group
Kennon	Heard		MD	University of Colorado	Denver, CO, USA	Site Investigator	Toxicology Investigators Consortium FACT Study Group
Robert	Hendrickson		MD	Oregon Health & Science University Hospital	Portland, OR, USA	Site Investigator	Toxicology Investigators Consortium FACT Study Group
Ziad	Kazzi		MD	Emory University Hospital	Atlanta, GA, USA	Site Investigator	Toxicology Investigators Consortium FACT Study Group
Eric	Lavonas		MD	Denver Health Medical Center	Denver, CO, USA	Site Investigator	Toxicology Investigators Consortium FACT Study Group
Michael	Levine		MD	UCLA- Olive View Medical Center	Los Angeles, CA, USA	Site Investigator	Toxicology Investigators Consortium FACT Study Group
Travis	Olives		MD	Hennepin County Medical Center	Minneapolis, MN, USA	Site Investigator	Toxicology Investigators Consortium FACT Study Group
Anthony	Pizon		MD	University of Pittsburgh Medical Center	Pittsburgh, PA, USA	Site Investigator	Toxicology Investigators Consortium FACT Study Group
Jonathan	Schimmel		MD	Mt Sinai Hospital	New York, NY, USA	Site Investigator	Toxicology Investigators Consortium FACT Study Group
Kapil	Sharma		MD	University of Texas Southwestern Medical Center	Dallas, TX, USA	Site Investigator	Toxicology Investigators Consortium FACT Study Group
Meghan	Spyres		MD	Banner University Medical Center	Phoenix, AZ, USA	Site Investigator	Toxicology Investigators Consortium FACT Study Group
Timothy	Wiegand		MD	Strong Memorial Hospital	Rochester, NY, USA	Site Investigator	Toxicology Investigators Consortium FACT Study Group
