# Supplementary material for: Identification of Bradycardia Following Remdesivir Administration Through the US Food and Drug Administration American College of Medical Toxicology COVID-19 Toxic Pharmacovigilance Project
Source: JAMA Netw Open. 2023 Feb 14;6(2):e2255815. doi: 10.1001/jamanetworkopen.2022.55815 (PMC9929701; doi:10.1001/jamanetworkopen.2022.55815)
Supplement: Supplement 3. — Data Sharing Statement [file jamanetwopen-e2255815-s003.pdf]

Data Sharing Statement

Devgun. Identification of Bradycardia Following Remdesivir Administration Through the US Food and Drug Administration American College of Medical Toxicology COVID-19 Toxic Pharmacovigilance Project. *JAMA Netw Open*. Published February 14, 2023.
doi:10.1001/jamanetworkopen.2022.55815

Data
Data available: No
